# Integration of Satellite Data with High Resolution Ratio: Improvement of Spectral Quality with Preserving Spatial Details

**DOI:** 10.3390/s18124418

**Published:** 2018-12-13

**Authors:** Aleksandra Sekrecka, Michal Kedzierski

**Affiliations:** Department of Remote Sensing, Photogrammetry and Imagery Intelligence, Institute of Geodesy, Faculty of Civil Engineering and Geodesy, Military University of Technology, 01-476 Warszawa, Poland; michal.kedzierski@wat.edu.pl

**Keywords:** data fusion, pan-sharpening, spatial resolution, spectral quality, Landsat

## Abstract

Commonly used image fusion techniques generally produce good results for images obtained from the same sensor, with a standard ratio of spatial resolution (1:4). However, an atypical high ratio of resolution reduces the effectiveness of fusion methods resulting in a decrease in the spectral or spatial quality of the sharpened image. An important issue is the development of a method that allows for maintaining simultaneous high spatial and spectral quality. The authors propose to strengthen the pan-sharpening methods through prior modification of the panchromatic image. Local statistics of the differences between the original panchromatic image and the intensity of the multispectral image are used to detect spatial details. The Euler’s number and the distance of each pixel from the nearest pixel classified as a spatial detail determine the weight of the information collected from each integrated image. The research was carried out for several pan-sharpening methods and for data sets with different levels of spectral matching. The proposed solution allows for a greater improvement in the quality of spectral fusion, while being able to identify the same spatial details for most pan-sharpening methods and is mainly dedicated to Intensity-Hue-Saturation based methods for which the following improvements in spectral quality were achieved: about 30% for the urbanized area and about 15% for the non-urbanized area.

## 1. Introduction

Data integration is an important and widely developed concept due to the easy access to a large number of different data [1,2,3,4], mainly including image data obtained from the satellite and aerial ceiling [5,6,7] and from low altitudes [8,9,10]. The concept of pan-sharpening, which refers to the fusion of satellite imagery, is popular in remote sensing. Such imagery is characterized by different properties and are usually assessed in terms of spatial and spectral resolutions. Most satellites can simultaneously acquire panchromatic (PAN) and multispectral (MS) imagery. Panchromatic imagery is a monochrome image, in which information collected from a wide range of the spectrum is stored in one channel. The spectrum range usually includes the visible range and a near-infrared portion of the spectrum (usually from about 0.45 µm to about 0.90 µm). The interpretation of imagery is, however, much more successful if there is access to multispectral imagery, in which data is collected in several narrow channels by detectors sensitive to a specific part of the electromagnetic spectrum. Such images allow for a more efficient recognition of objects, based on their different spectral properties. However, acquiring imagery from several spectral bands at once requires mounting more lines of detectors, compared to a panchromatic sensor, which only requires the use of one line of detectors. Consequently, the spatial resolution of a multispectral image is lower than the resolution of a panchromatic image acquired by the same sensor. A higher spectral resolution is obtained at the expense of spatial resolution [11]. Multispectral imaging can be applied in many fields of research such as: land-use and landcover mapping [1,12], archeological analyses [13], emergency response [14] and vegetation condition analyses [15,16,17]. This imaging often supports change detection [18,19] and also might provide information for predicting some phenomena [20]. The more spectral bands, the more objects can be distinguished. However, spatial resolution cannot be ignored here because it dictates what will be the smallest object possible to identify. Many methods have therefore been developed to integrate such images [21,22], which make it possible to generate a multispectral image with a spatial resolution of a panchromatic image. These methods are based on different approaches and, for this reason, can be divided into several groups. Two basic groups are component substitution-based methods (CS) and multiresolution analysis-based methods (MRA) [23,24,25]. CS methods are based on the projection of the multispectral image into another space. This transformation separates the spectral and spatial information which, in theory, should be recorded in different components [26,27]. These provide high spatial quality (the image is sharp), but then large distortions of colors appear [23,28,29]. This group includes primarily the Intensity–Hue–Saturation method (IHS) [30] and its extensions—the Generalized IHS (GIHS) [31], Principal Component Analysis (PCA) [32,33], Gram–Schmidt orthogonalization (GS) [34,35,36] and Brovey transformation [37], as well as methods which combine several methods, such as the Adjustable IHS and Brovey transform fusion technique GIHS–BT [31,38]. The issue of colour distortions is reduced in multiresolution analysis-based methods, starting from simple multiplicative methods [38], High Pass Filters (HPF) [39] and methods based on wavelet analyses [40,41] and ending with many more complex methods. These methods reproduce colours very well, unfortunately however, very often this is achieved at the expense of spatial quality. The pixel size of the sharpened image will be consistent with the spatial resolution of the panchromatic image, but the image will be blurred, and it will be difficult to identify the spatial details. Traditional methods extract the spatial details only from the PAN image without considering the structure information of the multispectral image and, thus, cause spectral distortion or deficient spatial enhancement [42]. Many modifications of pan-sharpening methods are proposed [33,43,44,45,46,47,48,49,50], therefore, which aim to increase the spectral quality and spatial integration of imagery data. Overall these methods give good results in typical cases when the data, which is being combined, had been obtained by the same sensor [51,52,53] and the ratio of spatial resolution is in the range of 1:2–1:5 [54]. Most often, for modern satellites with very high resolution, this ratio is 1:4. The spatial quality is at a level of 98% and spectral quality is at 70–90%, depending on the pan-sharpening method (assessed on the basis of the correlation with the panchromatic image after high-pass filtration and with the multispectral image, respectively) [38,55,56,57]. The problem of spectral distortions increases, especially for data obtained in various conditions by various sensors [11,55], which might happen when integrating high-resolution data with data from the Landsat series [8,58]. Regarding many analyses, it is desirable to recognize objects of the order of one or a few meters, which is guaranteed by VHRS (Very High Resolution) satellites, however, a simultaneous purchase of the PAN and MS image from a high-resolution satellite is usually very costly, which is often a limitation for some contractors. Currently, USGS provides free images from the Landsat series which, unfortunately, have a low spatial resolution (30 m in the multispectral range and 15 m in the panchromatic range). It is possible to increase this resolution by integrating low-resolution multispectral images with high-resolution panchromatic imaging with a resolution from several dozen centimetres to several meters. Additionally, there are still many systems which acquire only in the panchromatic channel and the injection of spectral information into them forces the integration of data recorded by various sensors, often with a different date of acquisition. Frequently, multispectral images from the Landsat series are used due to their wide availability. Any data set with a resolution ratio of more than the standard 1:5, will be considered as atypical for the purpose of this paper.

The problem of increasing spectral distortions when fusing atypical data comes, first of all, from data collection by detectors with different characteristics. Additionally, obtaining images from the same date and under the same conditions might be difficult to implement due to Landsat’s long revisit time (16 days) and the high degree of cloud cover of many images. Different characteristics of detectors and different conditions of image registration have a negative effect on the quality of image integration and can increase the spectral distortions of the image after the fusion process, mainly for vegetation and crop fields [11]. All the basic pan-sharpening methods, in this case, as well as all their modifications, will give worse results than in the case of a standard ratio of spatial resolution of the integrated images.

It is, therefore, necessary to develop an approach to improve the quality of the results of integrating satellite data with an atypical resolution ratio. Attempts to solve this problem have already been described in literature, where it is proposed to modify the panchromatic image to improve the spectral quality of the resulting image after pan-sharpening [59]. Then, satellite data integration takes place in two stages. The first stage is to modify the panchromatic image as the weighted average of the original panchromatic image and the intensity of the multispectral image, where the weights are dependent on the ratio of the amount of spatial information in each of the integrated images’ pixels. The second stage is to conduct pan-sharpening using any method. This approach makes it possible to enrich the spectral information of the panchromatic image, which further results in the improvement of the spectral quality of the image after fusion. The improvement of spectral quality alone, however, results in a decrease in spatial quality, which is a normal phenomenon and cannot be completely avoided. It is important to minimize this negative effect, especially for pixels displaying spatial details. The development of an approach to improve the spectral quality, while maintaining the original spatial quality in the vicinity of spatial details, is the subject of this article. It will be a desirable solution, especially when working with imagery of urbanized areas, both in terms of integrating high-resolution data with multispectral imagery as well as with hyperspectral data. The authors propose a simple solution which can be further developed in the estimation of weights by using, for example, alpha integration [60]. The least squares method can optimize the choice of weight values, while extending the process of processing big scenes.

## 2. Materials and Methods

### 2.1. Satellite Data and Study Area

Satellite imagery acquired by the Landsat 5 TM, Landsat 8 OLI/TIRS and WorldView-2 satellites were used for the purpose of this research. Satellites of the Landsat series are characterized by a Ground Sampling Distance (GSD) equal to 15 m for the panchromatic band, 30 m in the visible range and near and mid-infrared bands, and 60 m (Landsat 7) or 100 m (Landsat 8) in the infrared thermal range. Landsat 5 TM records imagery in seven bands, of which band 6 is a thermal channel. Landsat 8 has two sensors: OLI (registers in the visible range, near and mid-infrared-bands from 1 to 9) and TIRS (registers in thermal infrared-bands 10 and 11). Band 8 is a panchromatic channel, while band 9 is dedicated to cloud detection [61]. Table 1 lists further satellite bands and their spectral ranges.

Only bands in the visible, near and mid-infrared range, such as bands with a spatial resolution of 30 m, were used in the described studies. Concerning the Landsat 5 TM satellite data, the bands 1, 2, 3, 4, 5 and 7 were used, and for the Landsat 8 satellite bands from 1 to 7 were used.

WorldView-2 is a Very High Resolution Satellite (VHRS) and has a GSD of 2 m for bands in the visible, near and mid-infrared range, and GSD equal to 0.5 m for a panchromatic band. The first satellite band of the WorldView-2 is a panchromatic channel and has been used in this research as a high resolution image. The remaining bands record information in the visible and near- and mid-infrared range [62].

The research was conducted for several sets of satellite data. Each set consisted of a high-resolution panchromatic image and a low-resolution multispectral image. The data was selected to give the opportunity to examine integration results for various atypical cases. Dataset 1 consisted of a panchromatic image acquired by WorldView-2 and a multispectral image from Landsat 5 TM. The images were obtained under similar conditions. The image from the WorldView-2 satellite was acquired on 10 August 2010, and the image from the Landsat 5 satellite was acquired on 15 August, 2010. Dataset 2 consisted of the same Worldview-2 panchromatic image and multispectral images registered by Landsat 8 OLI on 23 April, 2015. These images were obtained with a big time range and during different seasons, which made it possible to examine the correctness of the method for atypical data. Both for Datasets 1 and 2, the ratio of spatial resolution was substantial and equal to 1:60.

**Figure 1:**
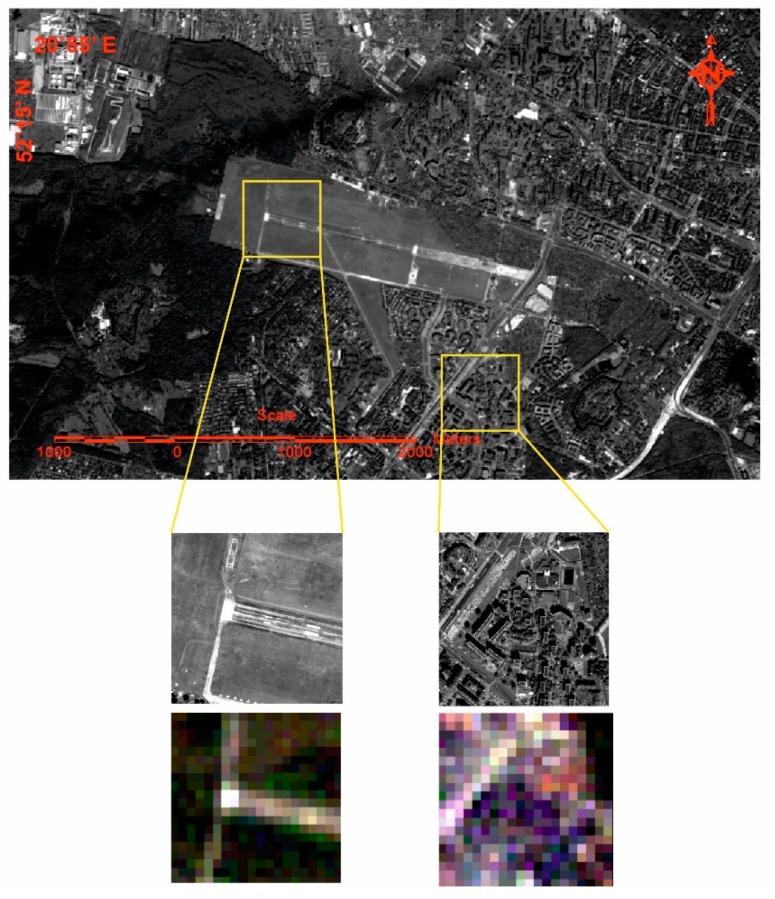
Study area—western Warsaw including regions of different types of land-use: panchromatic image form WorldView-2 (pixel size 0.5 m) and multispectral image from Landsat 5 TM (pixel size 30 m).

All studies were conducted for the same region, characterized by a diversity of land cover. The studied region was the western part of Warsaw, where there are both highly urban areas as well as non-urbanized, open areas (Figure 1).

The diversity of the forms of land-use in these images is characterized by different pixel values, which results from different spectral properties. Additionally, there is a significant difference in the image structure for open and urbanized areas. There are many different elements with different spectral properties in the urbanized area, which result in great variations in pixel values on the image. Regarding an open area with uniform terrain coverage, the pixel values are similar to each other. Thus, the studies carried out separate analyses for fragments of the images that show different types of land cover (Figure 1). The selected fragments of images will allow for testing of the integration results in terms of spectral and spatial quality for various types of objects.

The research used relative methods of assessing the quality of integration (based on a comparison of images after fusion with the original images). Consequently, the atmospheric correction process had been forsaken so as not to introduce additional distortions of pixel values and spectral properties. All transformations and analyses were carried out on the raw data.

### 2.2. Methodology of Data Integration

One of the methods to improve the spectral quality of data integration is the modification of the panchromatic image as a weighted mean of the panchromatic image with the intensity of the multispectral image, with the weights dependent on the ratio of spatial resolution of the integrated images [59]. The authors of this article propose to develop this approach, the values of the weights depending on the distance of the processed pixel from pixels containing significant spatial information. Such an approach is aimed at ensuring high spectral and spatial quality of the image after integration. The key task is to maintain the highest spatial quality, especially where spatial details occur. The extraction of such details from the panchromatic image is, therefore, one of the most important procedures. This detail extraction stage is very often buried within the algorithms of existing data fusion methods [38,39], which even more so emphasises the need to perform this step when modifying the panchromatic image to avoid false results.

**Figure 2:**
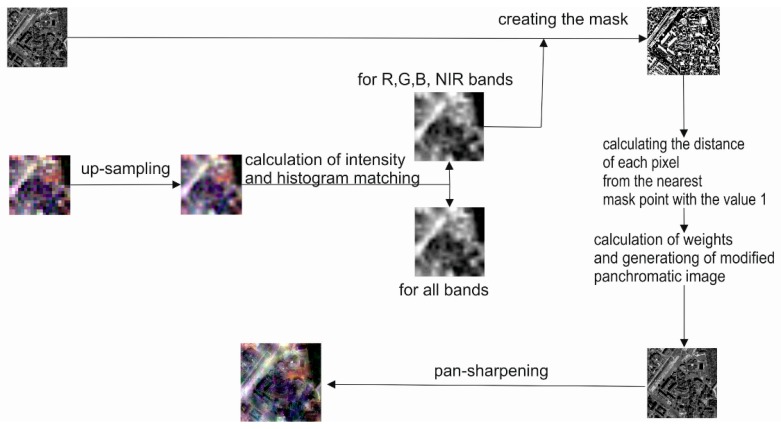
Diagram of the proposed pan-sharpening approach.

The sharpening of images, therefore, takes place according to the following stages (Figure 2):

- Multispectral image (MS) resampling to the panchromatic image resolution (PAN);
- Intensity calculation ($I_{MS}$) for the multispectral image, including all bands;
- Adjusting the intensity image histogram to that of the panchromatic image;
- Development of a mask showing those pixels, which illustrate the spatial details (information, which is not in the MS image);
- Calculation of the distance of each pixel of the image from the nearest pixel of the mask (from the nearest spatial detail);
- Determination of weights and calculation of a modified panchromatic image as a weighted average of the original panchromatic image and the intensity $I_{MS}$;
- Performing pan-sharpening by any method using the modified panchromatic image.

The sampling is necessary to correctly perform all calculations, because the size of each matrix must be the same. The modified panchromatic image is the weighted sum of the original PAN image and the intensity $I_{MS}$, therefore, to obtain the correct pixel values, these images should have previously matched histograms. The greater the weight of the panchromatic image, the greater the similarity between the original panchromatic image and the modified image, and the better the reconstruction of details, but with an insignificant improvement of the spectral quality of the integrated image. Using a high weight for the intensity image in turn will generate a modified panchromatic image with spectral properties similar to the multispectral image, but also significantly will reduce the spatial quality. The resultant image will be blurred, and it will be difficult to identify spatial features, which is contrary to the goal of pan-sharpening. Integration of different resolution data is performed to generate a new colour image that will make it possible to identify the same spatial details that can be recognized in a high-resolution panchromatic image. The key to this is the selection of weights, which will increase the spectral quality of integration results while maintaining high spatial quality.

#### 2.2.1. Proposed Method of Panchromatic Image Modification

The authors of this article propose that the value of weights be dependent on the presence of spatial details in close proximity to the modified pixel. As a result of an analysis, each pixel receives a value of 0 or 1. The value 1 is given to pixels that contain spatially significant information (these pixels are called “spatial details” in the article). The remaining pixels have the value 0. This creates a binary image (mask of spatial details, hereinafter referred to as a mask), which is the basis for further calculations.

Concerning each pixel of the image, an individual weight is calculated depending on its distance from the nearest spatial detail (from the nearest point, which has the value 1 on the mask). The weights are calculated in accordance with Equation (1):

(1) $$w_{1}=\frac{1}{e^{x}}$$

where $w_{1}$ is the weight for the original panchromatic image, *e* is the Euler number, *x* is the pixel distance from the nearest spatial detail.

The weight for intensity $I_{MS}$ is the complement of the weight $w_{1}$ to unity, as it is shown in Equation (2):

(2) $$w_{2}=1-w_{1}$$

**Figure 3:**
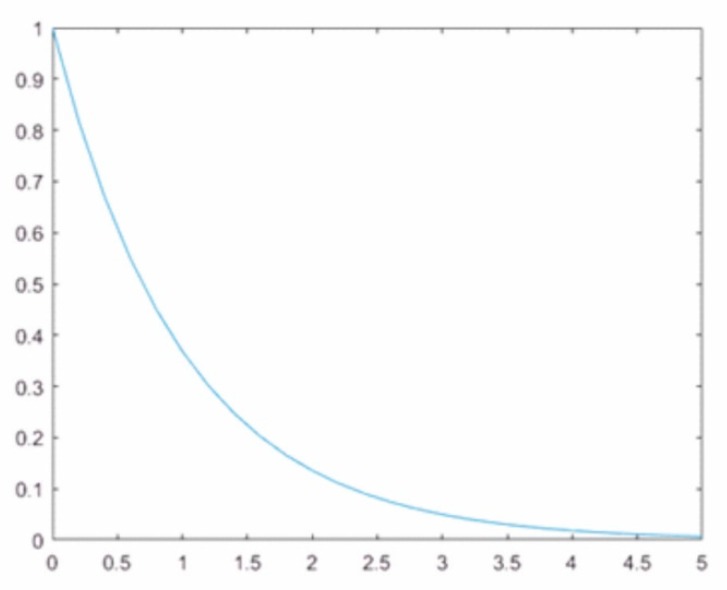
A plot of the function $\frac{1}{e^{x}}$.

**Figure 4:**
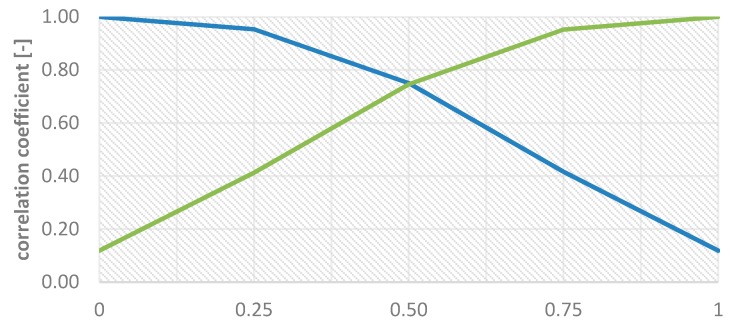
The correlation coefficient between the modified panchromatic image and the original panchromatic image (marked in blue) and the correlation coefficient between the modified panchromatic image and the intensity of the multispectral image (marked in green).

Considering the above equations and plotted Function (1) shown in Figure 3, it is easy to notice that the maximum value of $w_{1}$. might be 1 and minimum 0. A weight close to 1 will be assigned for the intensity image in areas where there are no spatial details, which means that the pixel values in the modified PAN image will only contain information from the multispectral image. Certainly, this definitely will increase the spectral quality of the image after fusion, however, any spatial information that could be injected from the high-resolution image will be lost, which undermines the essence of the pan-sharpening process. Therefore, 0.5 was taken as the maximum weight value and, after calculating the weights according to Equation (1), they are normalized so that the maximum weight value for the intensity does not exceed 0.5. Then, $w_{1}$ is contained in the range (0.5, 1) and $w_{2\;}$ in the range (0, 0.5), which guarantees a higher spectral quality while maintaining spatial quality. By analysing the graph (Figure 4), this is the greatest value at which the basic idea of pan-sharpening is still maintained.

The graph (Figure 4) shows the values of the correlation coefficient between the modified panchromatic image and the original panchromatic image (guaranteeing high spatial quality) and the correlation coefficient between the modified panchromatic image and the intensity of the MS image (guaranteeing high spectral quality). The functions intersect when both weights $w_{1}$ and $w_{2}$ are 0.5. There is the same degree of similarity between the modified image and the original high resolution image and the intensity in this case. Going beyond the value of 0.5 for $w_{2}$ significantly increases the influence of intensity in image modification, while the similarity to the high resolution image decreases. Correlation with the high resolution image at 60% is a poor result and produces a poorly sharpened image post-fusion. This image is not entrenched with enough spatial information sufficient to identify the terrain details.

#### 2.2.2. Proposed Method of Detail Extraction

The spatial details can be extracted from the image using various image processing methods. Typically, spatial details are structures that stand out from the background, so high frequencies can be observed on the boundary of such structures resulting from dynamic changes in pixel brightness. An area where there is a homogeneous background (pixel values are close to each other) is usually an area where there are no spatial details (for example a meadow). This property can be used to detect details by means of a wavelet transformation or high pass filtering in the spatial domain of the image. However, this approach allows for the detection of high frequency changes, such as only those on the edges (boundaries) of objects. The improvement of image quality can also be based on the algorithm for improving the contrast, dedicated for low-contrast images [63]. This algorithm allows better visibility of details, but also is based on edge detection.

**Figure 5:**
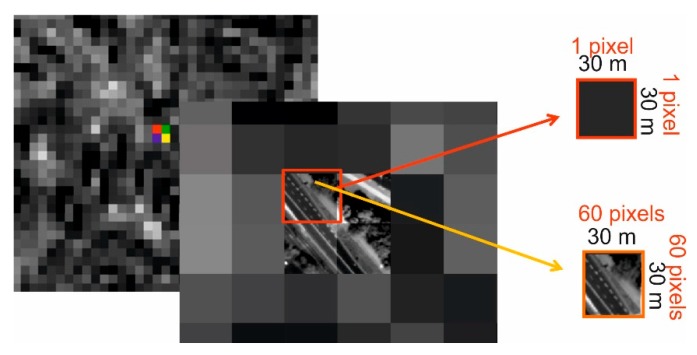
The relation between the pixel size of a panchromatic and multispectral image. One pixel of the multispectral image from the Landsat satellite (GSD = 30 m) corresponds to 60 × 60 pixels of the panchromatic image from the WorldView-2 satellite (GSD = 0.5 m).

The authors propose a different solution, where the detection of objects is based on the analysis of local histograms and binarization. Assuming that both images were acquired for the same area, one pixel of the multispectral image corresponds to *n* × *n* pixels of a panchromatic image, where n is the ratio of the pixel size of the low resolution image to the high resolution one. Considering the example of the data used in this study, one pixel from the Landsat satellite corresponds to 60 × 60 pixels of the panchromatic image from the WorldView-2 satellite (Figure 5).

Based on the above assumption, the PAN image can be divided into sub-matrixes (hereinafter referred to as blocks) corresponding to subsequent pixels of the multispectral image. Assuming that the images were acquired under the same conditions, the information stored in one pixel of a multispectral image is identical to the information stored in 3600 corresponding pixels of the panchromatic image. Thus, the average pixel value in a panchromatic block should be equal to or close to the intensity value corresponding to the pixel of the multispectral image. Ideally, the pixel average in the *k*-th panchromatic image block should be equal to the intensity of the respective pixel of the multispectral image (Equation (3)):

(3) $$\bar{PAN_{k}}=I_{MS}{}_{k}$$

where $\bar{PAN_{k}}$ is the average pixel value in the *k*-th PAN image block,$\;I_{MS}{}_{k}$ is the pixel intensity of the MS image corresponding to the *k*-th block in the PAN image. The intensity was calculated separately for each pixel of the MS image, which corresponded to the successive blocks of the high-resolution image. Because in reality it is not possible to obtain images in perfectly the same recording conditions, the average pixel value in a PAN image block is not always equal to the intensity of the corresponding MS pixel, and these values might differ by some difference $d_{k}$:

(4) $$d_{k}=\bar{PAN_{k}}–I_{MS}{}_{k}$$

The difference $d_{k}$ might result from different spectral ranges of the images to be integrated, or from different spectral properties of the objects (if dealing with a different growing season, etc.), and also from the various characteristics of the sensors that acquired these images.

**Figure 6:**
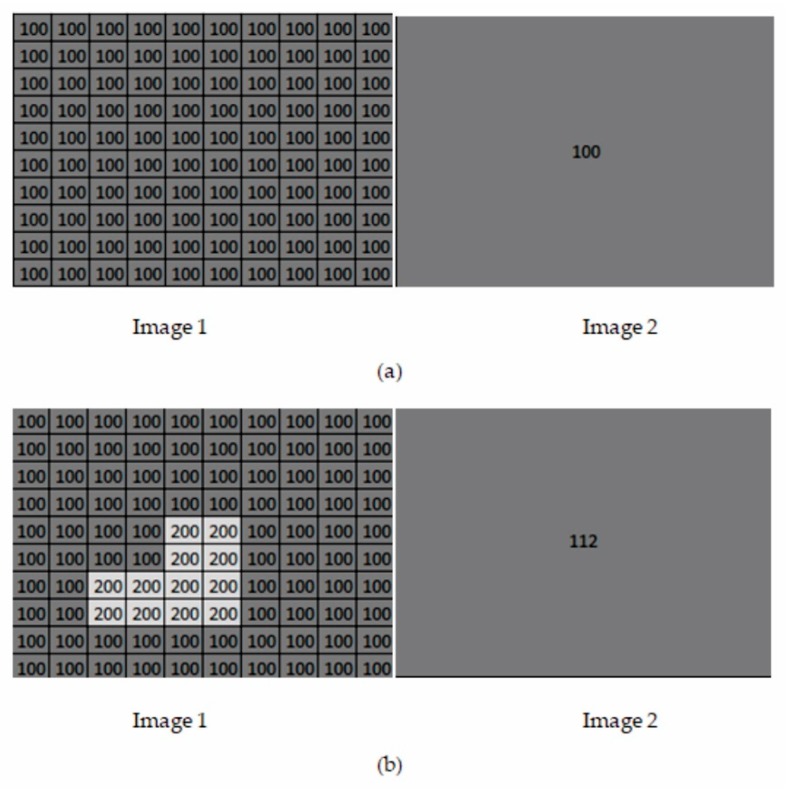
Sample images showing the idea of detail detection. Image 1 is an example of a high-resolution panchromatic image, Image 2 is an example of the intensity of a low-resolution, multispectral image. Two cases are shown: (**a**) there are no spatial details in the high-resolution image, (**b**) there are spatial details in the high-resolution image.

Equation (3) suggests that, if a tonally uniform homogeneous area was to be imaged, the intensity value of one pixel of the MS image and the average value of the corresponding pixels of the PAN image would be identical and each subsequent pixel of the PAN image would be the same as the pixel intensity of the MS image. Conversely, if a small object with different spectral properties appeared on this homogeneous area, then on a high resolution image it would be shown in the form of a group of pixels with different, dissimilar values. This can be considered in a simple example, where two images are given: Image 1 is a high-resolution panchromatic image and Image 2 is the intensity of a multispectral image with a resolution ten times lower. Looking at the first case (Figure 6a), a tonally homogeneous area is imaged (each pixel of the image has the same value equal to 100). Regarding both images, the information is averaged from the same area, thus, both the intensity of the multispectral image (Image 2) and the average pixel value of the high-resolution image (Image 1) is 100. The differences of the subsequent pixels between Image 1 and Image 2 are also 0.

Figure 6b shows the case when a bright object appears on that uniform background, so the pixel values showing this object in Image 1 are higher (the background has the value 100, and the object 200). The intensity of the low-resolution image will, of course, also slightly increase. Comparing the successive pixels of Image 1 with the intensity of the low-resolution image (Image 2), notice that in the area where the light object is visible, the differences between the PAN image pixels and the intensity of the MS are large. The remaining differences remain close to zero. The authors use this property to detect spatial details. To ensure the visual correctness of the resulting image, it is proposed to use an intensity image resampled to the resolution of the PAN image using bilinear interpolation.

Each subsequent pixel of the panchromatic image is then compared to the corresponding pixel in the intensity image:

(5) $$d_{i,j}=PAN_{i,j}-Ires_{i,j}$$

Ires denotes the intensity of the MS image after being resampled to the resolution of the multispectral image, i,j describe the position of the pixel, $d_{i,j}$ is the difference calculated for each subsequent pixel. As mentioned before, this approach is justified with ideally the same image recording conditions, which is practically impossible. It was mentioned in the example described above, (Figure 5), that values of differences $d_{i,j}$ close to 0 indicate that there is no spatial detail here. However, Equation (4) shows that the intensity of the MS pixel and the average value of the PAN image block might differ by a certain non-zero value and then such an assumption is unjustified. The value of $d_{i,j}$ should be compared with the value$\;d_{k}$, which will consider additional differences resulting from inhomogeneous conditions during image acquisition. When the difference between $d_{k}$ and $d_{i,j}$ exceeds a certain threshold, it is an indication of the occurrence of a pixel depicting the spatial detail. Using the proposed approach, only the difference value was significant, not its direction, therefore, when detecting spatial details, the absolute values of$\;d_{k}$ and $d_{i,j}$ were compared.

**Figure 7:**
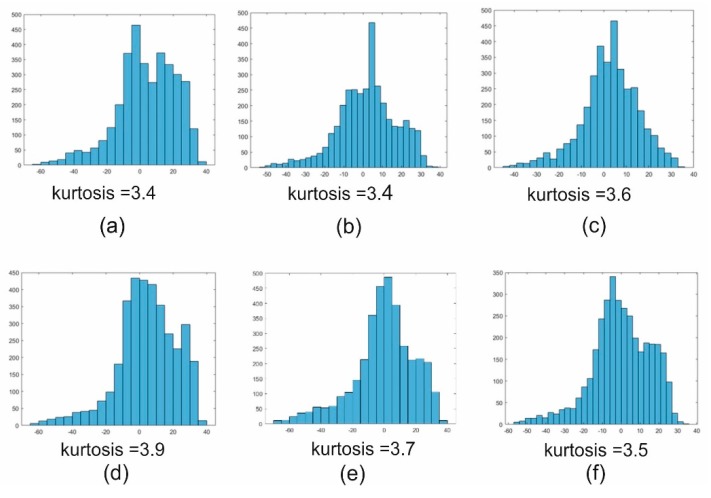
(**a**–**f**) Distribution of differences $\;d_{k}-d_{ij}$ in six sample blocks.

The threshold which classified pixels as spatial details was determined based on the statistics of the difference image $\left|d_{k}\right|-\left|d_{i,j}\right|$ in each subsequent block. Figure 7 shows the distribution of differences in six sample blocks.

An analysis of the distribution of differences in individual blocks shows that, in most cases, it follows a normal distribution (kurtosis is close to 3). The Gaussian distribution is characterized by a mean value and standard deviation. Based on this and previous analyses, it is assumed that the threshold for extracting the spatial details from the image will be a multiple of the standard deviation of the distribution of differences in the block.

A block’s statistics might be disturbed due to outliers. To ensure that correct statistics were being used, all outliers were eliminated, using double the value of the standard deviation as the criterion for removal. Then, a statistical re-analysis was carried out with the exclusion of outliers. The extraction of details is then based on such newly determined statistical parameters. When the value of the difference between each subsequent pixel of the difference image $\left|d_{k}\right|-\left|d_{i,j}\right|$ exceeded a given multiple of the standard deviation, then the pixel was classified as spatially significant. Figure 6 shows spatial details are imaged by pixels whose values differ greatly from those of the background. The authors use standard deviation to detect these details. The value of this multiplying factor depends on how many and how significant the details are needing identification. The possibility of detecting details for one, two and three multiples of the standard deviation were checked.

**Figure 8:**
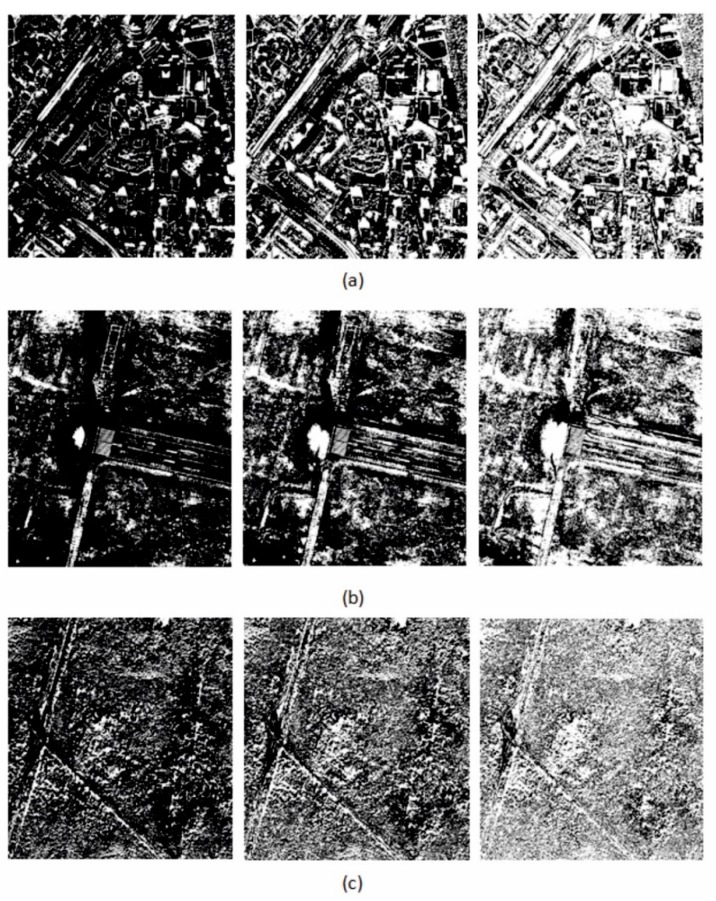
Detection of spatial details, assuming that the value of the difference $\left|d_{k}\right|-\left|d_{i,j}\right|$ does not exceed, respectively, 3 SD, 2 SD and 1 SD from left to right for a (**a**) high urban (**b**) open area (**c**) forest; where SD is the standard deviation of the differences in the block.

Figure 8 shows the results of the binarization process used to extract those pixels from the image, which include spatial details (white pixels). The threshold value was equal to the multiple of the standard deviation. Three threshold values were tested: 1SD, 2SD I 3SD. The lower the threshold value, the more details can be detected. The more details it is possible to identify, the greater the similarity will be between the modified panchromatic image and the original, and the lower the spectral quality measured for the entire image. Conversely, the more details will be kept, the higher will be the spatial quality. The choice of the threshold value is, therefore, dependent on the expected result. Three times the standard deviation allows the extraction of the details that differ the most from the background. Therefore, only those objects are extracted, which were much brighter or much darker than the intensity of the multispectral image. Bearing in mind the distribution of pixel values in an image, it can be said that, for a threshold of 3SD, only about 10% of the pixels will be classified as spatial details. Not all spatial details have such a high difference in pixel values, which is why many details are omitted at such a threshold. Using just one standard deviation, in turn, disregards a large number of pixels. Regarding the case of the analysed fragments, these pixels showed details that were of little importance (such as the different structure of a lawn), therefore, a multiple of two for the standard deviation was considered the optimal binarization threshold and further tests were carried out using such parameters. Considering this case, 30–40% of the pixels will be classified as spatial details, and this is usually the proportion of significant spatial information in the image. When comparing the image of the intensity of the MS image to the PAN image, one should bear in mind that they are acquired by different sensors, therefore, it is necessary to consider the resulting distortions. Thus, it is important that the images being compared are registered in similar spectral ranges. Panchromatic images are recorded over a wide range of the spectrum, covering the visible range and near infrared. Using the WorldView-2 satellite, this range is from 0.45 nm to 0.8 nm. The intensity of the multispectral image, used to extract details, was calculated according to the relationship (Equation (6)) for the four channels of the multispectral image corresponding to this range:

(6) $$I=\frac{B_{1}+B_{2}+\ldots +B_{n}}{\sqrt{n}}$$

where *I* denotes the intensity of the multispectral image, $B_{1}+\ldots +B_{n}$ are the subsequent bands of the multispectral image, within the visible and near infrared range, n represents the number of bands.

The method of detecting details based on statistics and spectral properties makes it possible to locate most of the spatial details. Other methods make it possible to detect only the edges of objects. The statistical method shows not only edges, but whole objects (especially objects of small size, which were highly reflective).

### 2.3. Methods of Assessing the Quality of Data Fusion

Integration of data with different resolutions is carried out using different methods and the question always arises which of the methods gives the best results. There is no unambiguous answer to this question, because the quality of integration can be considered in several aspects, the two most important of which are spatial and spectral quality. Spatial quality defines the similarity of the content of information about the spatial details between the image after the fusion and the panchromatic high-resolution image. Spectral quality measures the similarity between the content of spectral information recorded in the low-resolution multispectral image and the post-fusion image. Evaluation of the quality of integration can be carried out in two ways: quantitatively or qualitatively. Qualitative methods do not specify specific conditions, are subjective and are based on visual analysis. Quantitative methods, in turn, are defined by mathematical formulas that boil down to comparing the corresponding pixels in the images. Both visual and quantitative analyses were carried out in the presented research. The three properties defined by Wald’s protocol must be met [64,65,66] for the results of the quantitative assessment to be reliable:

- The first property (consistency property): any fused image, once degraded to its original resolution, should be as identical as possible to the original image;
- The second property (synthesis property): Any fused image should be as identical as possible to the ideal image that the corresponding sensor would observe with the highest spatial resolution, if existent;
- The third property (synthesis property): The multispectral set of fused images should be as identical as possible to the multispectral set of ideal images that the corresponding sensor would observe with the highest spatial resolution, if existent.

Therefore, to evaluate the fusion quality, the original panchromatic and multispectral images were degraded 60 fold in spatial resolution and the degraded images were pan-sharpened. The original multispectral image was used as reference for this quantitative evaluation.

The spectral quality assessment was based on the correlation coefficient, which is a measure of the similarity between the pixel values in subsequent channels of the image of the sharpened and original low-resolution multispectral image. It is calculated in accordance with Equation (7):

(7) $$CC_{\left(MS_{FUS},\;MS_{OR}\right)}=\frac{COV_{\left(MS_{FUS}\;MS_{OR}\right)}}{SD_{\left(MS_{FUS}\right)}\cdot SD_{\left(MS_{OR}\right)}}$$

where $MS_{OR}$ is the original MS image, $MS_{FUS}\;\mathrm{is}\;\mathrm{the}\;$ post-fusion MS image, *CC* is the correlation coefficient, *COV* is the covariance and *SD* is the standard deviation. The value of *CC* is dependent on the ratio between the covariance of the corresponding channels of both images and the spread of the pixel values around the mean value in each channel of both images (standard deviation). It follows, then, that for data that maintains ideally the same spectral properties, the correlation coefficient should be 1. The closer *CC* is to 0, the less correlated the compared data is with each other and, therefore, the less they are similar to each other [67].

To assess the image quality, the Structural Similarity Index (SSIM) also was calculated, which is the relation of mean values, standard deviations and correlations between the images [68]:

(8) $$SSIM=\frac{2\cdot M_{\left(MS_{FUS}\right)}\cdot M_{\left(MS_{OR}\right)}+C_{1}}{M_{\left(MS_{FUS}\right)}{}^{2}+M_{\left(MS_{OR}\right)}{}^{2}+C_{1}}\cdot \frac{2\cdot COV_{\left(MS_{FUS}\;MS_{OR}\right)}+C_{2}}{SD_{\left(MS_{FUS}\right)}{}^{2}+SD_{\left(MS_{OR}\right)}{}^{2}+C_{2}}.$$

To perform an assessment of the spatial quality, the Laplacian Index IL% [69] was chosen. It is based on the determination coefficient, which defines the similarity between the high-resolution image and the post-fusion image, which had both beforehand been sharpened using a Laplace filter [69,70]:

(9) $${IL}_{k}\%=100\%\cdot r_{K}^{2}$$

where $r_{k}^{2}$ is the coefficient of determination in the *k*-th channel of the post-fusion image. IL% is calculated for each channel separately, whereas for a global assessment, AIL% is proposed as the average value of IL% from all image channels [71]:

(10) $$AIL\%=\frac{1}{K}\cdot \overset{K}{\underset{k=1}{\sum }}IL_{k}\%$$

where K is the number of channels. The maximum value of IL% and AIL% is 1, which suggests that the same spatial information is shown on both images.

## 3. Results

The experiment included pan-sharpening for each dataset (described in Section 2) using the original panchromatic image (classical approach) and a panchromatic image modified in accordance with the approach proposed by the authors (modified approach). The data fusion was performed several times for each approach using several pan-sharpening methods (GIHS, GIHS-BT, HPF, PCA, Wavelet, Gram–Schmidt). The main aim of the research was to check to what extent the application of the modified panchromatic image improves the quality of integration results. The two methods which gave the best results were selected and used in the process of data fusion for Dataset 2 to investigate the effect of the modified panchromatic image on the quality of atypical data fusion.

A qualitative and quantitative assessment of the quality of the post-fusion images was made. During the spatial quality assessment, the entire post-fusion images were compared with the original panchromatic image, having all been previously sharpened with a Laplace filter to expose the spatial information. During the assessment of spectral quality, those pixels were considered, which had a value of 0 on the mask, meaning that their values had changed on the modified panchromatic image. Thus, a sample of 100 pixels was selected for each image subjected to the fusion and spectral quality indicators were calculated for this sample. This approach made it possible to assess the actual improvement in areas where it actually occurred. Additionally, the effect of the modified panchromatic image on the radiometric quality of the fused image was compared for the whole image by comparing the signal-to-noise ratio in the classic and modified approach.

### 3.1. Integration of WorldView-2 PAN and Landsat 5 TM

Table 2 presents the values of the correlation coefficient between the post-fusion images and the original multispectral image. 

The highest improvement in spectral quality (increase in the correlation coefficient) occurred after the use of the GIHS and GIHS-BT methods, mainly in the visible channels. Regarding the open terrain, the correlation coefficient increased in channels 1, 2 and 3, respectively, by about 22%, 7% and 11% in relation to the value from the classical approach. When applying the other methods, there was an improvement of about 2–6%. A higher increase in the coefficient is noticeable for the urbanized area, where, for the GIHS and GIHS-BT methods in channels 1, 2 and 3, the values increased by 20%, 10% and 17% respectively, while, for other methods, the spectral quality improved from 2% to 15% depending on the method and channel. The use of a modified panchromatic image for data fusion for images from Dataset 1 did not give good results for infrared channels, which is the result of different spectral resolutions of the data being merged.

Table 3 lists SNR, AIL% and SSIM values—mean values for all channels are given due to the high similarity between all values in each channel of the post-fusion image. The SNR values (Table 3) mainly increased by about 1–2 dB, which shows that the approach proposed by the authors does not deteriorate the radiometric quality of the post-fusion image, and might even cause its subtle improvement. The SSIM index values are very similar for both approaches. Values differ by no more than 0.02, which is a negligible difference. Such small changes result from the fact that modifications are subject to pixel variation, but the image structure does not change significantly. The ratio of statistics describing the structural similarity remains alike.

AIL% values decreased on average by about 12%, but in no case fell below 70%, which means a high level of spatial quality, which is sufficient to identify terrain details. It should be noted that the calculated metrics are based on pixel values and are, therefore, subject to some uncertainty regarding the assessment of the spatial quality of the fusion process. The improved spectral quality had to influence the brightness of pixels that were not classified as spatial details. However, each pixel carries a portion of the spatial information and any variation on its value will change the value of the spatial quality indicator. To reliably assess the spatial quality, a visual analysis was conducted, which made it possible to assess the practical changes in spatial quality in the image.

### 3.2. Integration of WorldView-2 PAN and Landsat 8 OLI

Based on earlier analyses, it was found that the modified panchromatic image, which had been modified using the proposed algorithm, gives the best results when using IHS based methods. Only the GIHS and GIHS-BT methods were used in further research to determine how the modified panchromatic image would improve the spectral quality of the atypical data (Dataset 2). Table 4 presents the values of the correlation coefficient for subsequent image channels post-fusion.

Regarding the case of Dataset 2 for the urbanized area, the correlation coefficient increased in each channel by about 30% in relation to the values from the classical approach, both for the GIHS and GIHS-BT methods. However, for the open area the results were different depending on the image band. The greatest improvement occurred in bands 4, 5, and 6, where information about red and near-infrared radiation is recorded. Concerning the GIHS method, the correlation coefficient increased in these bands by about 16%, while for the GIHS-BT method it was by 16%, 29% and 18% respectively for bands 4, 5 and 6. Looking at the other channels, the improvement was between 7–8% for the GIHS and GIHS-BT methods, respectively.

The signal-to-noise ratio does not change significantly, in most cases it is a subtle improvement of 1–2 dB (Table 5). Additionally, the SSIM values change only slightly, which confirms the reasoning from the previous dataset from Landsat 5 described above. The AIL% indicator shows, however, that the use of a modified panchromatic image causes a slight decrease in spatial quality (by about 3%) although it still remains above 95%, which means a very high spatial quality that makes the identification of spatial details possible, just as in a high-resolution image.

### 3.3. Visual Analysis

**Figure 9:**
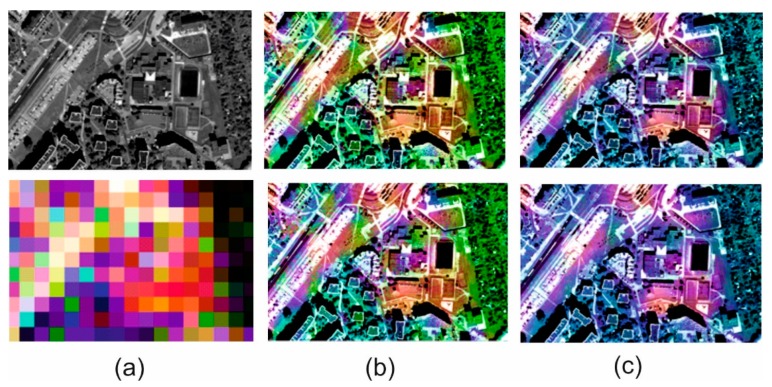
(**a**) Original panchromatic WorldView-2 image and multispectral Landsat 5 TM image. (**b**) Results of data fusion using the classical approach (above) and modified approach (below) after GIHS fusion. (**c**) Results of data fusion using the classical approach (above) and modified approach (below) after GIHS-BT fusion for urbanized area.

**Figure 10:**
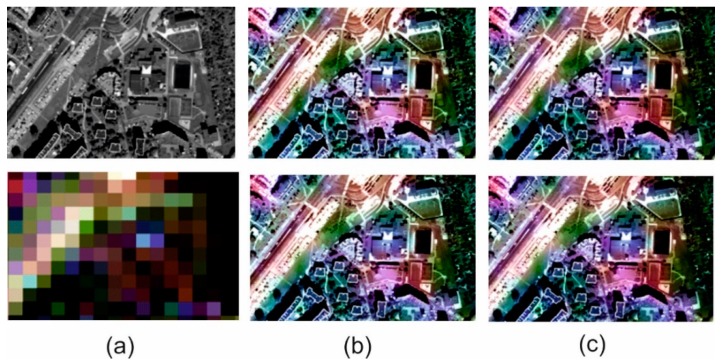
(**a**) Original panchromatic WorldView-2 image and multispectral Landsat 8 OLI image. (**b**) Results of data fusion using the classical approach (above) and modified approach (below) after GIHS fusion. (**c**) Results of data fusion using the classical approach (above) and modified approach (below) after GIHS-BT fusion for urbanized area

**Figure 11:**
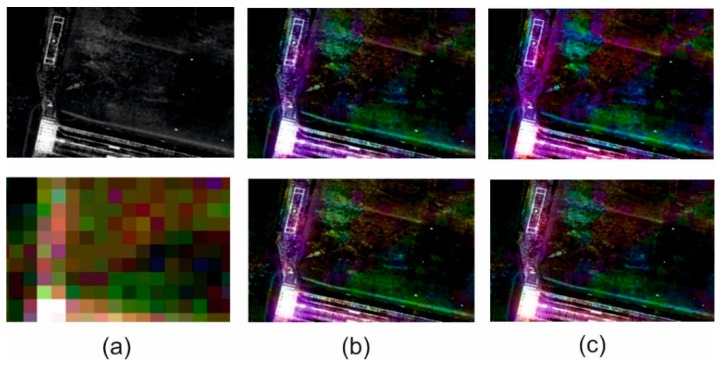
(**a**) Original panchromatic WorldView-2 image and multispectral Landsat 5 TM image. (**b**) Results of data fusion using the classical approach (above) and modified approach (below) after GIHS fusion. (**c**) Results of data fusion using the classical approach (above) and modified approach (below) after GIHS-BT fusion for open area.

**Figure 12:**
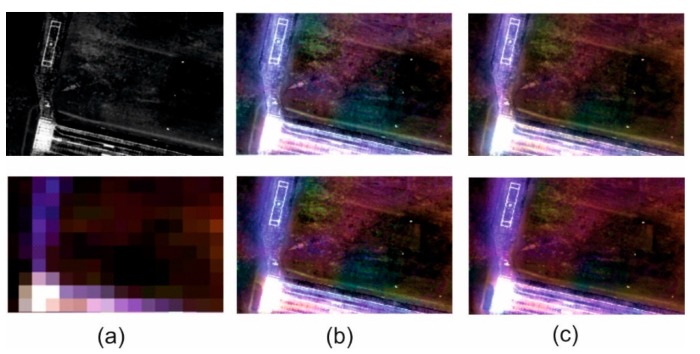
(**a**) Original panchromatic WorldView-2 image and multispectral Landsat 8 OLI image. (**b**) Results of data fusion using the classical approach (above) and modified approach (below) after GIHS fusion. (**c**) Results of data fusion using the classical approach (above) and modified approach (below) after GIHS-BT fusion for open area.

The spatial quality assessment indices are an indication describing the spatial changes in an image, but they are based on changes in pixel brightness and the results do not always match the actual interpretation possibilities in the image. Thus, quantitative analyses supported by qualitative and visual analysis where changes in the ability to recognize and identify subsequent terrain objects, have been performed for each image. Figure 9, Figure 10, Figure 11 and Figure 12 show results for pan-sharpening after applying the classic and modified approaches for both datasets for GIHS and GIHS-BT methods.

Visual analysis showed that the approach proposed by the authors did not disturb the spatial quality of integration with any of the methods studied. Subsequent to applying a modified panchromatic image to the fusion process, it is still possible to identify the same objects on the resultant images, which were visible after integration was done in accordance with the classic approach. Two groups of spatial details can be distinguished in the images: those that had the value 1 on the mask and those that had the value 0. The pixels belonging to the first group were not changed, so the spatial quality of integration in those areas did not change. The pixels with the value 0 on the mask have been changed to the weighted sum of the values of the corresponding pixels in the panchromatic picture and the intensity of the multispectral image based on the weights given by the Equations (1) and (2).

This has often caused local changes to the contrast in the post-fusion image, and the lowering of the contrast influenced, in turn, the deterioration of the possibility of automatic edge detection, which is the basis for calculating the metrics characterizing the spatial quality of integration. This gave weaker quantitative results in the assessment of spatial quality. However, visual analysis allows for elimination of this imperfection in mathematical methods and confirms that, despite locally deteriorated contrast, all the details of the terrain, both large and regular (buildings, roads) as well as narrow, small and irregular (paths, lines on the field, borders of various types of vegetation) can be recognized.

Visual analysis also makes it possible to verify the quantitative results of the spectral quality assessment of the image integration process. Spectral changes in the images shown above can be noticed, especially for the urban area. These changes are seen as local variations in pixel brightness and saturation. They occur in areas that were not classified as spatial details. Only the results of the GIHS and GIHS-BT methods have been shown in the figures, as in all of the other cases, as the quantitative indices also indicated these changes were subtle and also visually difficult to observe. The proposed methodology works most effectively with methods based on the IHS transformation, at the same time reducing the spectral distortions, which are common for this group of methods.

## 4. Discussion

The research described in this article confirmed that the fusion of atypical sets of image data might result in poor quality imagery, mainly in terms of spectral quality. What is more, the results of the integration depend on the character of the imaged terrain. Different results can be expected for open or urbanized areas. These conclusions are in line with the results described in other publications [58] where the quality of data integration of spectrally mismatched, high resolution ratio imagery data also was examined. Additionally, when comparing various pan-sharpening studies, they all show that the integration result is affected not only by input data but, above all, by the fusion method.

Moreover, the conducted research has shown that the approach proposed by the authors allows for the improvement of spectral quality, while maintaining spatially significant details. Although the indicators of spatial quality have deteriorated, this cannot be ruled out because it is a result of image processing. Despite this, the AIL% indicator was still over 70% for Dataset 1 and 95% for Dataset 2, which proves that a high spatial quality is maintained. Mathematical indicators are not the most accurate method of assessing the spatial quality and, therefore, this assessment was supported by visual analysis and interpretation by the operator, which showed that both the classic and modified approach can recognize the same spatial details. This is important mainly in built-up areas, where the contours of buildings, roads and lanes on the roadway or lines on a football pitch have been preserved in the original form without blurring.

What is also important, the modification of the panchromatic image for the purpose of data fusion, does not negatively affect the radiometric quality of the resulting images, and even causes their subtle improvement. Regarding both sets of data, in most cases the SNR values increased by about 1–3 dB.

However, when it comes to the spectral quality of the integration results, it generally improved quite significantly in those places that were not classified to the spatial details group, of course. Results for two types of land cover and two data sets were examined. One set was obtained under the same conditions, the second in a different growing season and with a large period of time between the two images. Concerning Dataset 1, the results of the image fusion were examined for several selected methods, which all showed an improvement, mainly in the visible range. The greatest improvement (up to around 20%) was achieved for methods based on the IHS transformation. Moreover, all the methods for Dataset 1 gave weaker results for infrared bands, which might be due to the misaligned spectral resolution of the data being integrated.

The spectral quality of data integration of Dataset 2 was two times lower than that of Dataset 1 but, after applying the modified panchromatic image, the quality improved by more, relative to the classical approach. The main difference occurred in the infrared channels where, for Dataset 2, there was an improvement of about 30% for the urbanized area and over 16% for the open terrain (for GIHS and GIHS-BT methods). Research has shown that the approach gives an improvement in spectral quality for each data set, regardless of whether they are obtained in the same conditions or not. It is important which sensors were used to obtain these data, however. The use of a modified panchromatic image allowed the improvement of the spectral quality in all channels for data acquired by Landsat 8, despite the images being acquired in different conditions.

Comparing the results from both datasets for different types of terrain, it is evident that the more diverse the image structure, the higher the improvement of the spectral quality. Therefore, the proposed approach is mainly dedicated to methods based on the IHS transformation and for imagery of urbanized areas.

Moreover, research has shown that the use of a modified panchromatic image to improve the quality of GIHS and GIHS-BT methods gives similar results. The spectral correlation coefficient in the modified approach for both methods was generally the same or very similar. Therefore, the use of panchromatic image modifications can improve simple methods without the need to further modify their algorithms, which is important considering the processing time for large images.

The research used raw data, without any atmospheric correction, because it was not necessary to investigate the correctness of the proposed approach. However, it should be remembered that by integrating data for specific spectral analyses based on a direct reference to ground data, this correction is necessary before performing any image processing. Examination of the approach on two different sets of data shows that for data with atmospheric corrections, the modification of the panchromatic image should increase the spectral quality of the post-fusion image also. A change of even just several percent is extremely important in such a case because it will better approximate the spectral properties of the objects extracted from the image to their actual reflection coefficients.

Work on similar topics already has been presented in the literature [59] where it also was proposed to modify the panchromatic image to increase the spectral quality of pan-sharpening. There, the key role was played by the weighting factor modulating the amount of information taken from both images to be integrated. This coefficient was the same for the whole image and, on the one hand, it ensured maintaining high spatial quality, while on the other hand, it limited the possibilities of improving the spectral quality. The approach presented in this article makes it possible to modify the panchromatic image to varying degrees depending on the area. Pixels are subject to modification, the size of which is inversely proportionate to the distance away they are from spatial details. The function given by Equation (1) guarantees a rapid increase of weights for the intensity of the multispectral image which, in turn, means that for the majority of pixels, the new value is close to the arithmetic mean of the values from the panchromatic image and the intensity image. This solution ensures high spectral similarity of the modified panchromatic image to the intensity of the MS image and, hence, a high degree of improvement in the spectral quality of the post-fusion image. Indubitably, the results are different for different methods but, studies have shown, that for the majority of commonly used methods such an improvement takes place, it can reach approximately 30%, compared to values from the classical approach. The article presents only research for several chosen pan-sharpening methods, but the results obtained suggest that, in many hybrid methods, one also can expect a high improvement in spectral quality because these methods are often based on the same basic solutions.

The approach proposed by the authors might be a new direction for the integration of multi-resolution and multi-sensor image data, both for satellite and aerial data obtained from low altitudes, as well as for terrestrial photogrammetry. The presented approach is based on pixel value analyses and works effectively, regardless of the spectral matching of the input data, which proves its versatility to be used in various fields and for different sets of data. The conducted research also showed some imperfections of mathematical indicators used to assess the quality of spatial integration and this suggests the need to develop a new approach to facilitate a more reliable assessment.

## 5. Conclusions

The conducted research has shown that the approach proposed by the authors makes it possible to improve spectral quality, while maintaining spatially significant details. This solution is especially dedicated to Intensity–Hue–Saturation-based pan-sharpening methods because they make it possible to obtain sharpened images with high spatial quality, but with spectral distortion. The research showed the greatest improvement in spectral quality for GIHS and GIHS-BT methods, while maintaining their spatial quality.

Additionally, the proposed approach gave the highest improvement (even up to 30%) for urbanized areas, where the image is characterized by a high variability of pixel values, which is a key issue when processing imagery of urban areas. Considering open, tonally homogeneous areas, the spectral quality increased from a few to a dozen or so percent, depending on the band of the image.

Moreover, the approach is dedicated to data integration of imagery with a large resolution ratio, which is accompanied usually by low spectral quality. Thanks to the panchromatic image modification described above, it is possible to increase this quality in all bands for data acquired by Landsat 8, despite the images being acquired in different conditions.

The article only presents research for the several selected pan-sharpening methods, but the results suggest that that in many hybrid methods one can also expect a high improvement in spectral quality, because these methods are often based on the same basic solutions.

The approach proposed by the authors might be a new direction for the integration of multi-resolution and multi-sensor image data, both for satellite and aerial data, data obtained from a low altitude, as well as for terrestrial photogrammetry. The presented approach is based on pixel value analyses and works effectively regardless of the spectral matching of the input data, which proves its universality for use in various domains and for various data sets. The conducted research also showed some imperfections of mathematical indicators used to assess the spatial quality of integration, which suggests the need to develop a new approach to facilitate a more reliable assessment.

## Figures and Tables

**Table 1:** Bands of Landsat7 ETM, Landsat 8 OLI /TIRS and WorldView-2 [61,62].

Landsat 5 TM		Landsat 8 OLI/TIRS		WorldView-2	
Band 1	0.44 µm–0.51 µm	Band 1	0.44 µm–0.45 µm	Band 1	0.45 µm–0.80 µm
Band 2	0.52 µm–0.60 µm	Band 2	0.45 µm–0.51 µm	Band 2	0.40 µm–0.45 µm
Band 3	0.63 µm–0.69 µm	Band 3	0.53 µm–0.59 µm	Band 3	0.45 µm–0.51 µm
Band 4	0.77 µm–0.90 µm	Band 4	0.64 µm–0.67 µm	Band 4	0.51 µm–0.58 µm
Band 5	1.55 µm–1.75 µm	Band 5	0.85 µm–0.88 µm	Band 5	0.58 µm–0.62 µm
Band 6	10.31 µm–12.36 µm	Band 6	1.57 µm–1.65 µm	Band 6	0.63 µm–0.69 µm
Band 7	2.06 µm–2.35 µm	Band 7	2.11 µm–2.30 µm	Band 7	0.70 µm–0.74 µm
		Band 8	0.50 µm–0.68 µm	Band 8	0.77 µm–0.90 µm
		Band 9	1.36 µm–1.38 µm	Band 9	0.86 µm–1.04 µm
		Band 10	10.60 µm–11.19 µm		
		Band 11	11.50 µm–12.51 µm		

**Table 2:** Correlation coefficient for the fusion of Landsat 5 TM and WorldView-2 imagery data using the classical (class) and modified (mod) approach.

Area	Method	Band 1		Band 2		Band 3		Band 4		Band 5		Band 6	
		Class	Mod	Class	Mod	Class	Mod	Class	Mod	Class	Mod	Class	Mod
open	GIHS	0.50	0.60	0.57	0.62	0.49	0.55	0.12	0.11	0.61	0.39	0.50	0.51
	GIHS-BT	0.49	0.60	0.58	0.62	0.49	0.54	0.12	0.10	0.61	0.39	0.50	0.51
	HPF	0.41	0.43	0.41	0.41	0.28	0.29	0.03	0.15	0.55	0.46	0.36	0.36
	PCA	0.40	0.41	0.36	0.41	0.25	0.26	0.11	0.18	0.50	0.33	0.35	0.36
	Wave	0.35	0.35	0.31	0.31	0.20	0.21	0.14	0.15	0.20	0.15	0.15	0.13
	GS	0.44	0.46	0.49	0.49	0.36	0.38	0.10	0.17	0.53	0.38	0.48	0.49
urban	GIHS	0.35	0.44	0.40	0.45	0.31	0.38	0.08	0.01	0.27	0.18	0.07	0.10
	GIHS-BT	0.35	0.43	0.41	0.45	0.32	0.38	0.07	0.02	0.27	0.18	0.06	0.09
	HPF	0.30	0.32	0.17	0.17	0.12	0.13	0.13	0.10	0.04	0.01	0.07	0.06
	PCA	0.44	0.52	0.48	0.49	0.43	0.43	0.17	0.19	0.21	0.11	0.15	0.21
	Wave	0.11	0.10	0.01	0.03	0.07	0.07	0.24	0.23	0.01	0.01	0.09	0.07
	GS	0.48	0.55	0.48	0.53	0.40	0.47	0.19	0.15	0.23	0.13	0.12	0.15

**Table 3:** Quality assessment indices for the fusion of Landsat 5 TM and WorldView-2 imagery data using the classical (class) and modified (mod) approach.

Area	Method	SNR [dB]		AIL% [-]		SSIM [-]	
		Class	Mod	Class	Mod	Class	Mod
open	GIHS	21.31	22.41	99.77	86.60	0.20	0.21
	GIHS-BT	21.25	22.18	99.74	86.32	0.21	0.21
	HPFA	24.18	24.50	99.77	86.81	0.18	0.17
	PCA	21.22	20.62	73.68	73.82	0.18	0.17
	Wave	23.72	23.90	84.48	71.79	0.11	0.10
	GS	21.25	21.27	99.77	86.80	0.23	0.22
urban	GIHS	13.99	14.72	99.97	87.78	0.15	0.12
	GIHS-BT	13.69	14.48	99.96	87.70	0.15	0.12
	HPFA	18.32	19.05	99.98	87.78	0.04	0.04
	PCA	19.19	19.51	94.14	83.87	0.10	0.09
	Wave	17.57	17.93	85.36	72.24	0.01	0.01
	GS	16.51	17.34	99.97	87.78	0.14	0.12

**Table 4:** Correlation coefficient for the fusion of Landsat 8 OLI and WorldView-2 imagery data using the classical (class) and modified (mod) approach.

Area	Method	Band 1		Band 2		Band 3		Band 4		Band 5		Band 6		Band 7	
		Class	Mod	Class	Mod	Class	Mod	Class	Mod	Class	Mod	Class	Mod	Class	Mod
open	GIHS	0.20	0.20	0.22	0.23	0.28	0.31	0.29	0.34	0.35	0.41	0.53	0.62	0.45	0.48
	GIHS-BT	0.20	0.20	0.23	0.24	0.29	0.31	0.30	0.35	0.34	0.41	0.52	0.62	0.45	0.48
urban	GIHS	0.24	0.35	0.24	0.33	0.25	0.36	0.24	0.33	0.18	0.27	0.21	0.29	0.18	0.28
	GIHS-BT	0.23	0.34	0.23	0.33	0.25	0.36	0.24	0.33	0.17	0.26	0.21	0.29	0.19	0.28

**Table 5:** Quality assessment indices for the fusion of Landsat 8 OLI and WorldView-2 imagery data using the classical (class) and modified (mod) approach.

Area	Method	SNR [dB]		AIL% [-]		SSIM [-]	
		Class	Mod	Class	Mod	Class	Mod
open	GIHS	22.01	24.38	99.77	95.68	0.13	0.12
	GIHS-BT	22.11	24.51	99.76	95.68	0.13	0.12
urban	GIHS	15.27	15.29	99.97	96.57	0.13	0.13
	GIHS-BT	15.15	15.13	99.96	96.55	0.13	0.13
